# The Human Sweet Tooth

**DOI:** 10.1186/1472-6831-6-S1-S17

**Published:** 2006-06-15

**Authors:** Danielle R Reed, Amanda H McDaniel

**Affiliations:** 1 Monell Chemical Senses Center, Philadelphia PA 19104, USA

## Abstract

Humans love the taste of sugar and the word "sweet" is used to describe not only this basic taste quality but also something that is desirable or pleasurable, e.g., *la dolce vita*. Although sugar or sweetened foods are generally among the most preferred choices, not everyone likes sugar, especially at high concentrations. The focus of my group's research is to understand why some people have a sweet tooth and others do not. We have used genetic and molecular techniques in humans, rats, mice, cats and primates to understand the origins of sweet taste perception. Our studies demonstrate that there are two sweet receptor genes (TAS1R2 and TAS1R3), and alleles of one of the two genes predict the avidity with which some mammals drink sweet solutions. We also find a relationship between sweet and bitter perception. Children who are genetically more sensitive to bitter compounds report that very sweet solutions are more pleasant and they prefer sweet carbonated beverages more than milk, relative to less bitter-sensitive peers. Overall, people differ in their ability to perceive the basic tastes, and particular constellations of genes and experience may drive some people, but not others, toward a caries-inducing sweet diet. Future studies will be designed to understand how a genetic preference for sweet food and drink might contribute to the development of dental caries.

## Introduction

**Figure 1:**
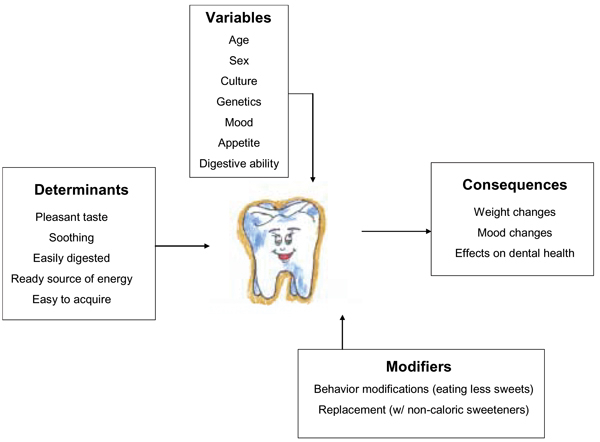
The determinants and consequences of the sweet tooth. The aspects that determine how humans respond to sweets are listed in the box labeled "Determinants". Individual variables that may influence these determinants are listed in the box under the heading "Variables". The box labeled "Modifiers" denotes those methods used to modify or change our attitudes and behaviors toward sweets. In the box labeled "Consequences" we list the physical and psychological effects of eating sweets.

The sense of taste gives us important information about the nature and quality of food, and of all the basic taste qualities, sweetness is the most universally liked. The human appetite for refined sugar and for sweet foods and drinks has been so strong that it has influenced the course of human history, and the recent and sharp rise in the consumption of sugar may be unprecedented. However, although there is a strong desire on the part of humans to seek and ingest sweet foods and drinks, it would be inaccurate to view the liking and enjoyment of sweetness and sweeteners as uniform across people and populations. The reasons for these individual differences are largely unknown, but the explanation for these differences could have important public health consequences. Several variables are good predictors of how well sweets are liked, such as age (*e.g*., children are more avid consumers than are adults). The term "sweet tooth" has been coined to describe people who "prefer" sweets, implying that these individuals differ from those who "do not prefer" sweets. However, sweet perception varies even in the same individual over time, and therefore a well-conceived model of the sweet tooth must be able to account for this observation and a broad range of other variables (Figure 1).

There are several facets of the human sweet tooth, and several academic disciplines, such as genetics, nutrition, and psychology, that contribute to our understanding of this trait. For instance, recent studies in mice demonstrate that DNA sequence variants in a sweet taste receptor gene have an effect on sweet preference and intake, and so it is possible that genetic differences among individuals may play a role in the human sweet tooth. Understanding sweetness also expands our understanding of bitterness. Bitter perception is related to sweet perception in humans, and these two sensory systems share some common features. The amount of calories we consume from sweet substances may play a role in the regulation of our body weight and overall nutritional status. Other variables help shape our behavior toward eating sweets and determine how many calories we consume from them, such as differences in our ability to digest sugars. Additionally, the taste of sweet may be sought for its pleasurable and soothing properties, and thus may be consumed irrespective of the nutritional consequences. Our individual behaviors toward sweets and our underlying reasons for consuming sweets are complex; therefore, an extensive understanding of the sweet tooth will assist us in tailoring nutritional advice.

### The Role of the Sweet Tooth

Some aspects of human experience are universally regarded as pleasant, and the experience of sweetness is a classic example. In many languages, the word for "sweet" connotes pleasant experience [1], and sugar has been introduced into many cultures where concentrated sweetness was unknown, with universal enthusiasm [2]. Our appetite for refined sugar and for sweet foods and drinks has been so strong that it has influenced the course of human history, and the recent and sharp rise in the consumption of sugar is perhaps unprecedented. This diet change has been the subject of popular books on the history and the culinary and biological effects of refined sugar [2-4].

An organizing explanation for the general attractiveness of sweets is that sugar has calories, and therefore sugar is a chemical signal for nutrients that can be readily detected and used for energy by animals. Furthermore, sugar also is a signal for safety because sweet items are rarely poisonous. Parenthetically, ethylene glycol, a chemical introduced into the environment by humans for use as antifreeze, is both sweet and poisonous and is responsible for a large proportion of deaths by accidental poisoning in dogs [5-7]. In nature, sweetness comes most often from honey and fruit and is processed from sugar cane and sugar beets. In some instances, sugar and sweetness are concentrated in the parts of plants that are meant to be eaten by animals so that the seeds can be dispersed, for example, dates or apples. Honey is an exception: it is an item that is meant to nourish bees but can be stolen by those willing to risk the consequences. Honey aside, highly concentrated sweet foods and drinks were not a part of the human diet until relatively recently. Today we refine sugar from sugar cane and sugar beets and also produce several artificial compounds to produce the sensation of sweet. The introduction of refined sugar into the lives of humans, first as a medicine and later as a food, has been full of consequences, not all of them desirable [8].

There is evidence to suggest that the liking for sweetness co-evolved with that for ethanol, because fermented fruit contains both [9]. Additional support for this hypothesis comes from the observation that ethanol and sweet perception share common neural substrates in rodents [10], and human alcoholics may have an increased sweet preference relative to those who are not alcoholics [11].

### Variation in Sweet Perception and Preference

Sugar is consumed because it tastes pleasant and is a ready source of energy that can be used by the body. Nevertheless, there is more variation than is widely appreciated in both perception and preference [12]. Thus, to get a better understanding of the variation seen in our response to sweet, we need to determine the relative contribution of our ability to detect and how intensely we perceive the taste of sweeteners versus our actual preferences for them. Additionally, we must consider that sweet taste perception and preference can vary both between individuals and in the same individual over a period of time.

#### Variation in detection and intensity

Individuals differ in their ability to detect the taste of sweet at low concentrations [13,14], and although rare, there are a few people who do not perceive a sweet taste from sucrose at all [15-17], a condition known as aglycogeusia. Likewise, the intensity ratings given for a single concentration of one sweet solution vary among people. The conscious perception of sweetness occurs when certain types of chemicals, such as sugars, contact taste receptor cells in the mouth. The taste receptor cell converts the chemical signal into an electrical one, and the nerve impulse is carried to the brain, where cortical taste areas are organized. Thus, the variation noted in the human ability to taste sweetness and the intensity with which it is perceived may be the result of differences in the taste receptor cells, in differences that occur along the pathway to the brain, or in the brain itself.

#### Variation in preference and desire

Aside from individual differences in our ability to detect and perceive the intensity of a sweet taste, there are large differences among people in the degree to which they like highly sweetened foods. Human subjects have been divided into two types. Type I responders are subjects who like increasing concentrations of sucrose up to a middle range of concentration, followed by a breakpoint after which preference decreases with increasing concentration. For these people, some foods and drinks are too sweet past a certain point, and this pattern is sometimes referred to as an inverted U-shape. The Type II response is characterized by increased liking as the concentration increases, which levels off but does not decrease as concentration increases further [18,19]. For these people, there is no such thing as too sweet. Although the classification of human subjects into two categories is probably too simple to capture the range of human responses to sweeteners, it highlights the dissimilarity among people.

#### Differences among groups

The perception and/or preference for sweet may vary not only from one individual to the next but also from one group to another. Studies have revealed effects of race, sex, and living situation (rural vs. urban). For instance, Americans of African descent prefer higher concentrations and Pima Indians prefer lower concentrations of sugar compared with those of European ancestry [20-25]. However, these racial differences may generalize only to specific food types. For instance, Taiwanese students rate sucrose solutions as more pleasant, but sweetened cookies as less pleasant, than do students of European descent [26]. Studies of sex differences suggest that male and female infants do not differ in sweet preference [20], but that older boys and men prefer higher concentrations of sweet compared with women [27,28]. Changes in the detection thresholds for sweet stimuli may tie into variations in sex hormone concentrations in women [29], but with conflicting effects on sucrose preference [30,31]. In addition to age and sex, the place someone lives may also influence sweet preference: Iranian children living in urban areas prefer much higher concentrations of sweetness in tea (a typical drink for these subjects) compared with children in rural areas [32].

#### Change over time

Variations in the perception of sweet may also occur within the same individual over time. These changes may be short term (*e.g*., changes in internal states) or long term (*e.g*., changes related to development and age).

Our need for sugar may dictate our perceptions and/or preferences for sweet, and day-to-day or periodic changes in our internal states may change this need. For example, the bodily fluctuations associated with our state of satiety that dictates our need for calories may influence our preference for sweet. When metabolic changes occur that reduce the availability of glucose in the blood, such as increases in plasma insulin concentration, then sweet preference increases [33-36]. Other longer-term changes to energy homeostasis in the body, such as weight gain or weight loss, may also alter our perception and/or preferences for sweet (*e.g*., [37,38]. Leptin, a hormone secreted by adipocytes, is a signal to the brain to indicate high or low energy reserves [39], and its action both in the brain and on taste tissues may influence our need and preferences for energy-dense foods, including sweets (further discussed below under Biology and Genetics of Sweet Taste). Additionally, other types of internal states may alter our perception and/or preferences for sweets. Sweet has been purported to share neurochemical pathways with alcohol and other drugs stimulating endogenous opiates [40,41]; therefore, shifts in psychological states (*e.g*., periods of depression, emotional upset, or anxiety) may increase our sensitivity to and craving for the rewarding properties of sweet taste [42,43].

Factors that produce more or less permanent modifications in our perception and/or preference for sweets may be the result of changes that occur as we develop and age. For instance, children are especially avid consumers of sweet foods and drinks. Babies are born liking sweet foods and drinks, as witnessed by facial expressions that display contentment and enjoyment [44,45]. Children prefer a higher concentration of sucrose in solution compared with their mothers, and this effect is true for at least two racial and ethnic groups [46], and perhaps applies to most or all cultures. However, the liking for concentrated sweetness fades during adolescence [21,47]. Although the reasons for this change are not known, they may have to do with changes in caloric requirements for growth, or the onset of puberty.

Overall, it is important to note that perception and preference are distinct measures. They may be related; for instance, in the simplest case, if someone could not perceive sweet taste (*e.g*., if granulated sugar tasted like sand), we would not expect that person to seek and consume sweet foods and drink. Thus, in studies measuring preference for sucrose at low concentrations, it is important to consider that some people will not be able to perceive the stimuli as well as others. However, perception and preference may also be unrelated except in the most extreme cases of perceptual loss. Thus far in human studies, sweet detection threshold does not predict either how intensely higher concentrations are perceived or how much they are liked [48-51]. In mice, there is a clear relationship between peripheral sensitivity and intake of sweeteners [52]; however, it appears that this relationship in humans is less clear.

### The Study and Measurement of the Sweet Tooth

To gain a thorough understanding of both the differences and similarities among individuals in the perception of sweetness, and to form a more substantial model for determining its use as a nutrient, scientists have performed numerous studies and employed many different measures. The study of the human sweet tooth has been undertaken by psychophysicists who measure sensory thresholds and intensity ratings, psychologists who study the desirability and hedonic and addictive qualities of sweetness, molecular and cellular biologists who seek to understand the transduction machinery that turns a chemical signal (sugar in the mouth) to an electrical signal that is decoded in the brain, and nutritionists and physicians who study why people choose sweet foods as nutrients and the effects of this choice on health. The emphasis on sweet perception is motivated to no small extent by the almost drug-like desirability of sweetness, with the goal often being to find out how to give people an experience of sweetness without the negative consequences of too many calories and tooth decay.

#### Methods to study sweet perception in humans

Most tests of sweet perception use sugar dissolved in water as taste stimuli. However, tests can use foods or complex solutions [53,54]. The results of these tests are related to the type of food or drink tested, so, for instance, one person might prefer a lower concentration of sweetness in tomato soup and a higher concentration in a fruit-flavored drink, whereas another person might prefer the reverse. Whether one uses sugar dissolved in water or a more complex stimulus to measure sweet perception, laboratory stimuli may only partially generalize to other sweet foods and drinks and to behavior outside the laboratory [55-57].

Measurement of the sweet tooth can be categorized in the following way: (1) assessment of sensitivity, as in detection/recognition thresholds, perceived intensity, and quality description; and (2) hedonic assessment, as in pleasantness, preference and craving. Tests of taste sensitivity measure the ability to taste the stimulus and determine its quality, and tests of taste hedonics are designed to measure how pleasant the stimulus is and the desire to consume it. Sometimes both of these types of measures are used in a single study to gain a more comprehensive understanding of what factors contribute to the sweet tooth [58,49]. Also, sometimes DNA is collected and analyzed together with psychophysical and behavioral measures to uncover the genetics of sweet taste perception [57]. Additionally, researchers have used many of these tests in studies designed to understand what role the sweet tooth plays in nutritional status, and determine if it may be a contributing factor in disorders such as diabetes and obesity [59,60].

Threshold tests measure the lowest concentration of a stimulus that a subject can detect or recognize [61]. In intensity tests, subjects are often asked to rate how intense a range of sweet concentrations is perceived to be, often relative to a standard. Quality tests require that the subject determine whether the stimulus is sweet, salty, sour, bitter, or savory. Sometimes these various measures are combined into one test. For instance, when measuring detection and recognition thresholds, the subject is asked to determine at what concentration he or she can detect the stimuli and then again at what concentration he or she is able to distinguish or "recognize" the taste as "sweet." Detection thresholds are almost always lower than recognition thresholds, because most subjects are able to determine if there is something in a solution before they can identify its quality.

There are several types of hedonic measures for taste stimuli. In one type of test, people individually rate a taste stimulus (*e.g*., sucrose) for pleasantness [62-66,19] or liking [67,49,68]. In other tests, subjects are offered two or more stimuli and asked to pick the one preferred [23]. Sometimes investigators ask subjects to rate a stimulus as being sweeter or less sweet than their ideal conception of what the stimulus should taste like [53] or ask them to rate themselves in terms of their level of sweet preference [69].

Learning how infants and children respond to different taste qualities both perceptually and affectively is a harder task because they simply do not have the language skills or the cognitive ability to relay their responses verbally or with paper-and-pencil tests. Therefore, to obtain data from young children, scientists rely on facial responses or other nonverbal responses. Facial responses are specific to certain taste qualities, and to a trained professional it is possible to decipher a facial expression that denotes pleasure versus pain, or one elicited from the taste of a sweet versus a bitter or sour stimuli [70-73] (reviewed in Erickson and Schulkin) [74]. These types of tests have also been used to look at the affective responses to tastes and smells in other animals, such as rats [75] and some types of primates [73,76]. The use of nonverbal measures of sensory perception or preference is not limited to children. Facial expressions are a useful addition to the study of adults [77]. Likewise, to get a more objective measure of physiological responses to taste stimuli, some investigators have studied heart rate changes or other responses not under voluntary control, such as sweating [71]. The use of these measures may partially circumvent the desire on the part of the subject to give a socially desirable response.

Understanding the sweet tooth also requires looking at the genes that are responsible for our ability to perceive the taste of sweet and the variations in these genes that contribute to individual differences in responses to sweet. Generally, scientists will ask subjects to perform psychophysical or behavioral tests and at the same time collect DNA. DNA can be collected from blood or from buccal cells that line the insides of the mouth, which is a good choice because it is less invasive than collecting blood [78]. DNA can then be genotyped to detect variants either in specific known genes (*e.g*., genes with a known involvement in sweet taste) or at a certain location along a chromosome that is predicted to harbor a gene involved in sweet taste. Once DNA is genotyped, researchers can ascertain whether responses on psychophysical or behavioral tests correlate with specific genotypes [57].

Nutritionists, physicians, and others interested in how the consumption of sweets influences health incorporate many of these perceptual and affective indices into their studies to help them understand why people choose to eat sweet foods and to determine if there are nutritional consequences to these choices [79]. There are various studies that examine the relationship between body weight and thresholds for sweet taste perception and/or preferences for sweets, reviewed in McDaniel and Reed, 2004 [80]. Some experiments test whether there is a correlation between body weight and the foods people eat, including sweets and fats, and also examine the concomitant effects of weight on thresholds and/or preferences for sweet stimuli. Others have people of various weights (*e.g*., underweight, normal weight, overweight, and obese) ingest a preload of a caloric substance and then have them rate their perception and/or preference for sweet stimuli. These tests help determine if there is in an interaction between immediate energy available as calories (preload) and long-term energy stores (body fat) that may influence sweet perception. Studies such as these help scientists and others understand the effects of body weight on the perception of sweet taste and, reciprocally, how sweet taste perception and preference may influence body weight.

To gain a more thorough understanding of how sweet perception and preferences interact with body weight, we need to establish how they translate into actual appetitive behaviors. Food diaries or questionnaires are useful in determining when, why, and where people choose to consume the foods that they eat [81,82]. They are also useful because they are a real-world measure, unlike one taken in the artificial setting of the laboratory. However, due to the disinclination of subjects to report all the foods they consume correctly [83], especially ones they may consider "naughty," such as highly sweetened candies and cookies, other proxy measures of sweet consumption can be used to evade reporter bias, such as the measurement of dental caries [42,84].

These psychophysical and behavioral tests may be used with other indices, such as the measurement of appetite and weight-regulating hormones, to determine the physiology underlying the effect of body weight on the perception, preference, and appetite for sweet foods [33,85]. Combining these measures assists researchers in understanding the physiological mechanisms that are linked to our appetitive behaviors.

Although all of the above-mentioned measures have generated much knowledge on the sweet tooth, scientists must also be able to account for all variables that may influence how a subject responds on tests and the validity of these results in a real-world setting. As already noted, responses vary when taste tests are conducted on several different occasions [86,87], although these changes in sweetness perception and preference are small compared with, for example, changes in olfactory threshold over time [88]. In addition to subject characteristics (*e.g*., ancestry, sex, and age), factors such as time of day, hunger, recent change in body weight, hormonal fluctuations, and testing conditions alter sweet perception and, therefore, need to be taken into account (for a review, see McDaniel and Reed, 2004)[80].

It is also important to remember that, although measures of sweet preference using sugar solutions are commonly used in the laboratory, they may not predict the preference for other sweeteners besides sugars [89] or selection of sweet foods or beverages at mealtime. Investigators have tried to bridge the gap between preference measures for laboratory stimuli and preference measures for foods and drinks by using mixtures of sugar and milk [54,90] or by adding sugar to simple beverages or foods [53]. As observed from comparing self-reported eating behaviors, such as food diaries, with sweet preference measures inside the laboratory, there is not always complete agreement [23,56].

Each type of test – threshold, perceived intensity, quality description, and hedonic – may measure both overlapping and unique perceptual processes that differ among individuals. Studies in which all of these measures are made in the same subjects are rare, and the extent to which individual differences in threshold, the ability to detect small increases in concentration, or perceived intensity affect pleasantness ratings has not been comprehensively studied. The studies to date have suggested these relationships are weak or non-existent [49,91,92]. However, the selective study of people at the extremes of sensitivity, high and low, may be needed to determine whether these relationships exist and how important they are for human sweet consumption.

### Biology and Genetics of Sweet Taste

The initial events in the perception of sweet taste in humans occur in taste receptor cells on the tongue and palate, which are found clustered in taste buds in taste papillae. Taste papillae on which most sweet receptors reside can be seen on the tip of the tongue and look like raised pink bumps or nipples (from which they derive their Latin name), and the perception of sweet intensity is related to their density [93,94]. Inside the taste papillae, taste receptor cells produce proteins that participate in sweet taste transduction, and some of these proteins are inserted into the cell membrane to form taste receptors. Taste receptor cells within taste buds contain neuropeptides that may modify and modulate the activity of neighboring cells [95]. In mice, taste receptor cells synapse onto primary afferent fibers, and the signals are relayed to the gustatory cortex via the nucleus of the solitary tract, parabrachial nucleus, and thalamic gustatory area, with a concentration of sweet-activated neurons in the rostral fields of these regions [96].

In the human taste bud, some cells express sweet receptors and respond to sweetness, whereas other cells express bitter receptors and respond to bitter chemicals. If a taste receptor cell is changed so that one that normally would be sensitive to sweetness instead expresses a bitter receptor, the animal treats that particular bitter chemical as though it were sweet. This result indicates that the brain reads the input of certain cells on the tongue as sweet, regardless of how those cells are stimulated [97]. In fact, even in response to primary taste qualities, the pattern of individual brain activity is consistent when one person is measured on several occasions, but people vary from each other, with each person's pattern being nearly unique [98].

Inside the taste receptor cell, two proteins combine to create a sweet receptor [99,100]. The names of these proteins are T1R2 and T1R3, for taste receptor family 1, proteins 2 and 3, and the names of the associated genes for these proteins are TAS1R2 and TAS1R3 [101]. In humans, if T1R3 pairs with the first member of this family, T1R1, the receptor is sensitive to compounds such as monosodium glutamate that are described as savory or *umami*, a Japanese word that translates as "delicious." The gene coding for T1R3 was discovered with the aid of mapping experiments in mice. Inbred mouse strains differ in their intake of saccharin and other sweeteners, and the results of experiments in breeding suggested that an allele of a single gene was partially responsible for these differences [101]. Then, through positional cloning approaches, this gene was identified and found to be *Tas1r3 *[99,102-106].

Alleles of the mouse *Tas1r3 *gene are related to differences in the consumption of sweeteners [107]. Recordings of the peripheral taste nerves suggest that mice with the low-preference *Tas1r3 *alleles exhibit lower nerve firing in response to saccharin or other sweeteners [52,108]. Removal of the *Tas1r3 *gene in mice results in a diminished response to most sweeteners [109], and when both genes, *Tas1r2 *and *Tas1r3*, are removed or "knocked out," the response to sweeteners disappears [110]. The results of mouse gene knockout studies disagree about the degree to which mice lacking these receptors learn to drink sweeteners if given periods of exposure to sweetened water. However, in mice unaccustomed to sweetened water, those with removal of one or both sweet receptors do not consume these solutions in preference to water. Likewise, domestic cats and tigers do not prefer sweet solutions compared with water. This lack of interest in sugar is probably because they cannot taste sweetness because the DNA sequence of one of the relevant receptor genes (TAS1R2) has decayed over time [111].

If DNA sequence variants have a large effect on the intake of sweeteners in mice and cats, then it is possible that this may also be true in humans. Because the peripheral neural responses of humans to sugars predict their verbal reports about sweet intensity [112], peripheral differences in taste sensitivity may be a component of human behavior toward sweetness. Cell-based assay systems have been developed to study the function of sweet receptors, and in experiments where the DNA sequence of the genes were modified, the results suggest that small changes in the specific amino acid sequence of in the human T1R2 and T1R3 protein can lead to differences in intracellular signaling in response to sweeteners [113,114]. These experiments, taken in conjunction with individual differences in the psychophysical responses to sweeteners [115-117], suggest that alleles of these receptors might influence sweet perception. A full range of human variation in sweet perception probably exists [12,118,119], and genetic variation in taste receptor genes may account for some or many of these person-to-person differences.

How genetic differences in sweet perception or liking that might exist in humans might translate into food intake and food preference is unclear. A review of the literature concluded that, although there was evidence that the degree of liking or preference for individual food items, including sweet items such as candy or sugared coffee, was genetically determined, the pattern of macronutrient consumption, such as whether the person ate more carbohydrate than fat, was more heritable [120]. Since the publication of that review, two studies have reported that families demonstrated significant genetic linkage for sugar intake to several chromosomal locations [121,122], one of which is near a sweet receptor gene (*1p36*). Overall, there is modest evidence that genetic differences among people might account for a portion of individual differences in the consumption of sweet foods and drinks.

There are explanations for person-to-person differences in taste function other than genetic differences. For instance, hormones modify taste receptor cell activity and differ in concentration among people. Leptin is one example: although its receptors are located primarily in the hypothalamus, several peripheral sites of action have been discovered. One such site is on the taste receptor cell itself, and studies in mice have demonstrated a role for leptin in suppressing the physiological and behavioral responses to sweets [123]. Such a response was noted in a study of the differences in preference for sweet solutions between wild-type (nonmutant) mice and the *db/db *mouse, a genetic mutant lacking a functional copy of the gene that codes for the leptin receptor [124]. Mice were administered exogenous leptin, which, through its action on the sweet taste receptor, suppressed the neuronal activation to sweet stimuli in normal mice, which subsequently reduced their consumption of sweet solutions. However, neuronal activation was not suppressed in the *db/db *mutant mice, and their consumption of sweets was not diminished [125].

As demonstrated in other studies that employ mice as model organisms, the extrapolation of mouse data to humans must be approached with caution. Despite this note of caution, further examination of whether differences in leptin concentration or activity might influence sweet perception and/or preference is warranted. Although one study found no relationship between plasma leptin concentration and sweet preference in obese women [85], the manipulation of plasma leptin concentrations in humans and their consequences for the perception and preferences for sweets may be a logical next step in unraveling how the body uses the perception of taste to regulate energy consumption.

Hormones and neurotransmitters that participate in higher-order cortical brain systems that organize the conscious perception of sweet or that regulate the rewarding properties of sweet may further serve to control our reaction to sweet chemicals (for a review, see Levine *et al*., 2003)[126]. Family studies of addiction have demonstrated that alcoholics and their family members prefer sweeter solutions than do nonalcoholic family members, suggesting that alcohol and sweeteners may share similar reward pathways in the brain [11]. The hypothesis that the rewarding properties of opioid-receptor-stimulating drugs and sweet taste are controlled by a common neural substrate is upheld further by the ability of naloxone, an opioid antagonist, to reduce both the positive properties of opiates and the preference for the taste of sweet [127,128]. Scientists are currently investigating alleles of genes that code for opioid receptors to determine if these alleles may be associated with the sensitivity to the rewarding properties of sugar and also to the suppressive abilities of naloxone.

### Bitter/Sweet Perception

The perception of sweet has had an extensive role in our evolution, but no more so than the taste of bitter. Vegetables contain many toxic compounds and are often bitter as a consequence, although humans have been working to breed out the bitter components and enhance the sweet components of our foods since selective plant breeding and human agriculture began. Plants cannot run from predators and so have developed chemical defenses that work by causing illness when eaten: by reducing enzyme activity, irritating the mouth and gut, provoking an allergic response, or altering hormone metabolism [129]. Early humans learned to heat plants to the point that many bitter compounds were less bitter and less toxic, and to add sweeteners to bitter foods. Both practices have expanded the human food supply.

Sweetness is used to mask the bitterness of medicines, and one French aphorism summarizes despair as "an apothecary without sugar." Some aspects of bitter perception by humans are caused by genetic variation [130-133]. Children with a genetic sensitivity to some bitters report that they like more highly concentrated sweetness compared with children who are less genetically sensitive to bitter [57]. Therefore, individual differences in bitter sensitivity may influence sweet intake, especially in children.

### Our Behavior Toward Sweets and the Consequences

The term "sweet tooth" has been coined to describe the preferences of a person who "likes" sweets, but it is less clear whether simply liking sweets can predict the tendency to consume more sweets. Our actual behavior toward choosing to consume sweet foods is harder to decipher and may stem both from our liking and desire for their taste and from the environment and culture in which we live. Furthermore, our behaviors toward sweets have consequences for our health, and these consequences provide feedback that could serve to either reinforce or alter our future behaviors toward their consumption.

As described earlier in this chapter, there are a variety of different responses to the taste of sweet, and these surely play a role in our motivation to eat sweets. However, another reason that sugar may be consumed is for benefits other than taste (*e.g*., digestible calories), and people might differ in their ability to utilize or benefit from calories in sugars (*e.g*., hereditary sugar intolerance) [8,134]. Because there are several types of naturally occurring sugars and enzymatic steps associated with the specifics of their digestion, people vary in their ability to digest sugars. A difficulty in digesting sugar may, consciously or unconsciously, change behavior toward sugar. Some people may even begin to find the taste of sweet unpalatable, because they begin to associate this taste with the negative digestive consequences caused by eating sweet foods [135]. This behavioral effect is called a conditioned taste aversion [136]. These behavioral changes may, in turn, have consequences on health. For example, people with hereditary fructose intolerance learn to avoid fructose; as a consequence, they often do not have dental caries or other secondary problems related to sugar consumption. About 10% of the native inhabitants of Greenland do not tolerate sucrose [134], but the consequences for diet and dental caries prevalence in this group are not known.

Behaviors toward sweets may also be formed by our psychological need for the rewarding properties of sugars. Sweets may alleviate depression and premenstrual symptoms and provide relief from the cravings for other drugs, because sweet taste releases opiates into the blood [126]. The pacification provided by sugar and sweets has been demonstrated to increase the liking of sweets [137] and may also translate into a behavioral tendency to consume more sweets. Studies that have examined the intake of sweets in women with psychiatric problems have reported that their consumption is increased compared with those without reported psychiatric problems [42]. Overall, sugar is consumed because of its pleasant taste, ease of digestion, and positive effect on mood, and these three reasons are not mutually exclusive; each probably makes a contribution to overall behavior.

People's behaviors toward sweets are also biased by circumstance and by their beliefs about the food or drink they taste. If people are told they are drinking a particular brand of soft drink, they will have a different pattern of brain activation compared with the activation seen when they drink the same drink but are not told the brand name, or if they are told the beverage is a different brand name [138]. Although the chemical stimulation from the beverage is the same, previous experience and the person's beliefs about a particular brand alter the response of the brain as the beverage is tasted. A humorous example of the power of learning and experience comes from a study of newly married women from Oaxaca, Mexico, who reported a change in their sweet preference after marriage that brought them closer to the preference of their mother-in-law [139]. Thus, learning from our families and culture whether sweets are either acceptable or unacceptable may influence our behavior toward liking and consuming them and may have consequences for our health.

Our willingness to consume sweet nutrients is also probably influenced by supposed or known dietary effects that we have encountered personally, been advised of by health professionals, or read in popular literature. The highly caloric nature of most nutrients that contain sugars has led many individuals to avoid or restrain their intake of such substances. In addition, adverse effects on dental health and the threat of other undesired health-related conditions may quell the desire to eat sweets. Thus, despite the almost universal liking for the "taste" of sweets, many find the idea of consuming them in excess to be "bittersweet." However, although a stigma toward excess sweet intake may exist, the actual negative effects of eating sweets are less clear and have been debated in the experimental literature.

One such perceived negative effect on health is the fear that excess sugar consumption leads to obesity. However, studies on the effects of sweet intake on body weight have yielded conflicting evidence, some demonstrating a negative correlation between sweet intake and body weight and some reporting the opposite, a positive correlation between sweet intake and body weight (for a review, see Hill and Prentice, 1995) [140]. Studies that have measured food intake outside of the laboratory have mostly reported that lean subjects consume more of their total calories from sugar than overweight or obese subjects. This is not surprising given that some heavier people may be restraining their intake of foods that they consider more "fattening." However, proxy indices of sugar intake, such as the number of dental caries lesions, conflict with this finding, indicating that overweight women actually eat more sweet foods than do lean women [42].

Research on the biochemistry of energy expenditure reveals the specific metabolic consequences of eating different food types, including sugars. In contrast to fats, sugars are biologically "expensive" to store and are therefore burned as calories before fats [141]. Thus, if we contrast a diet high in sugars to a diet high in fat, a sugar-rich diet would seem to indicate a higher rate of metabolism and produce a leaner person. However, our daily food intake is more complex, and many foods we consume that are high in sugar are also high in fat. In fact, according to one study, high sugar content improves the palatability of foods high in fat, which may cause one to consume more fatty food [54]. In addition, the rewarding pleasurable qualities of sweet foods may lead some to consume sweet foods in excess of dietary requirements, to self-soothe. Furthermore, the state of the food (*i.e*., liquid or solid) may play an additional role in appetitive behavior. In studies where subjects are asked to add sugar as a liquid supplement to their diets, they do not appear to compensate for the additional calories by decreasing their intake of other food sources [142]; however, when sugar is introduced into the diet as a solid, there is a subsequent decrease in the amount of additional calories that are consumed [143]. It appears that our choice to eat sweet foods either as a replacement or as an addition to other nutrients may be a more correct predictor of whether sweet foods will have an effect on body weight than are mere measurements of total sweet intake. Thus, both the attitudes we have toward eating sweets and our actual eating behaviors are important in our regulation of body weight.

Another well-known consequence of consuming excess sugars is the negative impact on dental health [144]. When sugar is consumed, particularly sucrose, naturally occurring bacteria inside the mouth interact with the sugar and produce acids that demineralize enamel on teeth. This demineralization process creates dental caries (lesions on teeth), which produce pain and, if left untreated, will erode and destroy teeth. The amount and severity of dental caries are positively correlated with sugar intake, and particularly with extrinsic sugars (*e.g*., sugars that are refined and added to foods). For example, foods such as bakery confections, including cookies, candies, and cakes, are much more cariogenic than are foods containing intrinsic sugars, such as milk and fruits. Some have concluded that sugar is no longer a large threat to dental health because of the addition of fluoride (a remineralizing agent) to drinking water and to toothpastes. However, studies have revealed that, even in those receiving treatments of fluoride, a strong positive correlation remains between high sugar consumption and the prevalence of caries. Nevertheless, the combination of fluoride and lowered sugar intake does appear to have a synergistic effect on the reduction of dental caries. Keeping this in mind, it is important to note that dental caries is a progressive chronic disease that, through preventative measures such as lowering sugar intake and brushing with fluoride, can be stopped or at least delayed for long enough to prevent severe complications.

### Modifying Our Sweet Tooth

To combat some of the either real or perceived problems with a sugar-laden diet, researchers have been looking for ways to modify our sweet tooth to reduce sugar intake. One way to reduce our need and intake of sugar is by replacing it with high-intensity sweeteners. We have been adept at harnessing a diverse array of chemicals that have a sweet taste but that contain few calories [145]. Because the use of low-calorie, high-intensity sweeteners by humans has a short evolutionary history, the long-range consequences of uncoupling sweetness from calories and introducing new food additives are unclear. One concern is that oral sweetness, for example from aspartame-sweetened gum, prepares the body for calories that it does not then receive and thus may exacerbate hunger [146]. Another concern is that high-intensity sweeteners may cause cancer or other negative health effects.

An alternative route to reducing the negative effects of sugar intake has been to attempt to reduce or enhance sweet preference through experience early in life. Many people, parents particularly, try to reduce the sugar preference of their children by reducing their children's intake of sweets, especially in infants and very young children. However, animal studies that have tried to reduce sweet liking by changing earlier experiences with sugar have thus far been unsuccessful [147-149]. Whether children who eat little sugar in childhood will like sugar less as adults is not known.

## Conclusion

The sweet tooth is culturally universal and has played an important role in human evolution. However, the perception of sweet differs greatly across individuals and groups, and behaviors and preferences toward sweet are affected by an entire range of variables, from genetics and age to personal experiences and cultural beliefs. Studies of sweet perception have increased our understanding of individual differences, and genetic studies are expanding our knowledge even more. Finding ways of combining different types of measures into single studies gives us a more comprehensive understanding of the sweet tooth and the role it plays in our lives, including the consequences on our health. We hope, with continued and focused study, to unravel more of the mysteries that surround our sweet tooth so that we may find more effective ways to shape and modify it for our well-being.

## Competing interests

The author(s) declare that they have no competing interests.

## Authors' contributions

All authors read and approved the final manuscript
